# Antibacterial mechanism of hyper-branched poly-L-lysine against methicillin-resistant *Staphylococcus aureus* and its synergistic and antagonistic interactions with conventional antibiotics

**DOI:** 10.3389/fmicb.2025.1676135

**Published:** 2025-10-08

**Authors:** Yaoqin Wang, Qiaoli Wu, Guofeng Mao, Lulin Rao, Meichun Liang, He Sun

**Affiliations:** Department of Clinical Laboratory Center, Shaoxing People’s Hospital, Shaoxing, China

**Keywords:** methicillin-resistant *Staphylococcus aureus*, hyper-branched poly-L-lysine, antibacterial activity, antibacterial mechanism, fractional inhibitory concentration index

## Abstract

The global proliferation of multidrug-resistant pathogens, particularly methicillin-resistant *Staphylococcus aureus* (MRSA), constitutes a severe threat to public health, rendering many conventional antibiotics obsolete. In the post-antibiotic era, where the pace of bacterial resistance evolution far exceeds that of new drug discovery, novel therapeutic strategies are urgently required. This study investigates the antibacterial potential of hyper-branched poly-L-lysine (HBPL), a synthetic antimicrobial polymer, against MRSA. We elucidate a multi-modal, physically disruptive mechanism of action initiated by electrostatic binding to the bacterial envelope, followed by rapid membrane permeabilization and cellular collapse, as visualized by electron and confocal microscopy. HBPL demonstrated potent, concentration-dependent bactericidal activity against clinical and standard MRSA strains, with a minimum inhibitory concentration (MIC) of 0.5 mg/mL and a minimum bactericidal concentration (MBC) of 1.0 mg/mL. Furthermore, HBPL inhibited biofilm formation by three MRSA strains (87, 73, and 81%) at a concentration of 1.0 mg/mL, which is a great advantage against the persistent and recalcitrant nature of biofilm-associated infections. Combination therapy studies using checkerboard assays revealed mechanistically dependent interactions. A synergistic effect [fractional inhibitory concentration index (FICI) ≤0.5] was observed with levofloxacin, attributed to HBPL-mediated membrane permeabilization enhancing intracellular drug access. Conversely, antagonism (FICI > 4) was noted with daptomycin, likely due to competitive binding at the bacterial membrane. These findings underscore the importance of rational drug pairing. While prolonged exposure induced stable, low-level resistance in MRSA, this was associated with adaptive cell envelope remodeling rather than target-site mutation. Collectively, this research establishes HBPL as a promising membrane-active agent and adjuvant therapy, capable of not only direct bactericidal action but also of restoring the efficacy of existing antibiotics against formidable pathogens like MRSA.

## 1 Introduction

The dawn of the 21st century is shadowed by the escalating crisis of antimicrobial resistance (AMR), a global health threat that jeopardizes the cornerstones of modern medicine (Liu et al., 2024). The Global Research on Antimicrobial Resistance (GRAM) Project, in a landmark analysis published in *The Lancet*, estimated that AMR was directly responsible for 1.27 million deaths in 2019, a figure exceeding the annual mortality from HIV/AIDS or malaria (Naghavi et al., 2021). *Staphylococcus aureus* has remarkable genetic plasticity which has enabled it to acquire resistance to nearly every antibiotic class introduced into clinical practice (Vahabi et al., 2014). The emergence and global dissemination of MRSA epitomize this challenge. This pathogen’s ability to combine multidrug resistance with potent virulence factors makes the development of novel anti-MRSA strategies a paramount objective for global public health (Touaitia et al., 2025).

The clinical challenge of MRSA extends far beyond genetically encoded resistance mechanisms. Two key survival strategies—biofilm formation and the generation of persister cells—create a veritable fortress that shields the pathogen from both host immunity and antimicrobial therapy (Vanamala et al., 2021). Biofilms are structured communities of bacteria encased in a self-produced extracellular polymeric substance (EPS) matrix, which adheres to both biological surfaces and medical implants (Touaitia et al., 2025). Embedded within these biofilms, and also present in planktonic populations, is a subpopulation of dormant, metabolically inactive bacteria known as “persister cells” (Kim et al., 2018). These cells are not genetically resistant but exhibit phenotypic tolerance to antibiotics because the drugs’ targets—active processes like DNA, protein, or cell wall synthesis—are downregulated or inactive (Korczak et al., 2024). Upon cessation of antibiotic therapy, these persisters can resuscitate, leading to infection relapse (Kim et al., 2018).

In the search for novel antimicrobials, nature has provided a compelling blueprint in the form of host-defense peptides (HDPs), also known as antimicrobial peptides (AMPs) (Basso et al., 2020). AMPs exhibit broad-spectrum activity against bacteria, fungi, and viruses, primarily through a mechanism that involves electrostatic interaction with and physical disruption of microbial cell membranes (Tajer et al., 2024). However, despite their promise, the translation of natural AMPs into clinical therapeutics has been fraught with challenges, including high manufacturing costs, susceptibility to proteolytic degradation, and sensitivity to physiological conditions such as pH and salt concentration, which limit their stability and bioavailability (Basso et al., 2020).

To overcome the limitations of natural AMPs, researchers have turned to the design of synthetic antimicrobial polymers that mimic their essential features while offering superior stability and scalability (Santos et al., 2016). Cationic polymers, in particular, have emerged as a highly promising class of therapeutics (Si et al., 2022). These macromolecules are engineered to present a high density of positive charges, enabling them to electrostatically target the net-negative surface of bacterial cell envelopes (Alkarri et al., 2024). Following this initial binding, the polymer’s amphipathic nature facilitates its insertion into the lipid bilayer, leading to membrane destabilization, pore formation, leakage of cellular contents, and ultimately, rapid bacterial death (Si et al., 2022). The versatility of polymer chemistry allows for the precise tuning of molecular architecture, charge density, and hydrophobicity to optimize antibacterial efficacy while minimizing toxicity to mammalian cells (Carmona-Ribeiro and de Melo Carrasco, 2013).

Among the diverse architectures of antimicrobial polymers, we focus on HBPL, a synthetic polymer constructed from the amino acid L-lysine. The hyper-branched architecture provides a compact structure with a high concentration of terminal primary amine groups, which are protonated at bacteric physiological pH to confer a strong cationic charge. The use of L-lysine as the monomeric unit is a key design feature, imparting excellent biocompatibility and potential biodegradability, which are critical for medical applications (Deepak et al., 2024). Previous work has demonstrated the potential of HBPL in various biomedical contexts, such as in the development of antibacterial coatings for medical devices and as a component of advanced wound dressings, establishing its promise as a versatile antimicrobial biomaterial (Tajer et al., 2024). Despite the demonstrated potential of HBPL, a comprehensive understanding of its fundamental antibacterial mechanism against a clinically paramount pathogen like MRSA remains incomplete. Furthermore, its capacity to act as an adjuvant in combination with conventional antibiotics—a critical strategy for combating multidrug resistance—has not been preliminarily investigated. This knowledge gap hinders the rational design of HBPL-based therapies.

**Figure 1:**
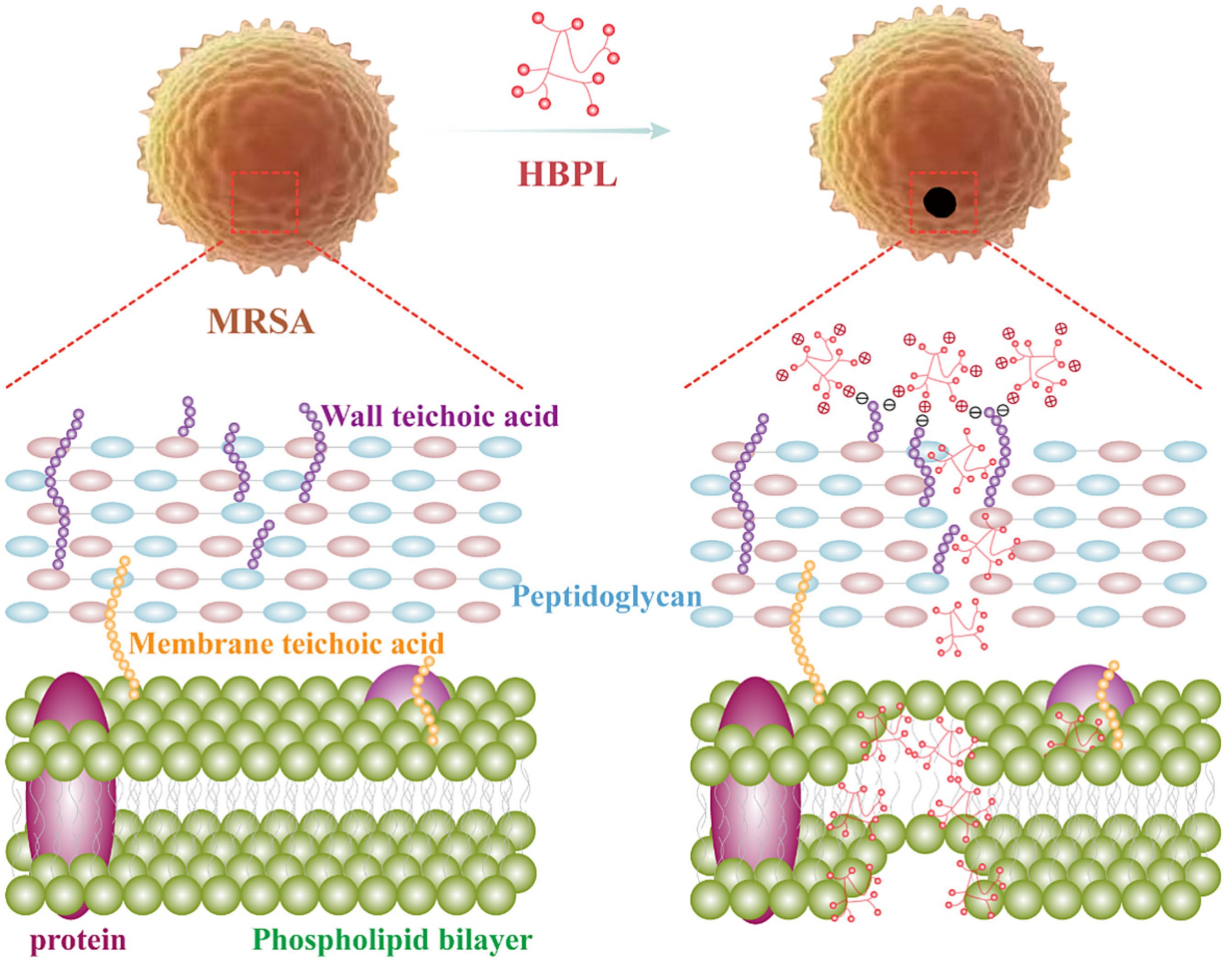
Schematic illustration of the antibacterial mechanism of HBPL against MRSA.

In this study, we elucidated a multi-modal, physically disruptive antibacterial mechanism of HBPL against MRSA: the positively charged HBPL binds to the negatively charged MRSA cell surface via electrostatic attraction. Following adsorption, HBPL penetrates and disrupts the bacterial membrane, leading to pore formation and eventual cell lysis and death (Figure 1). HBPL has also been demonstrated to effectively against biofilm formation, which was benefited to against the persistent and recalcitrant nature of biofilm-associated infections. These findings showed HBPL’s potential as an antimicrobial agent against infections caused by MRSA. Notably, HBPL exhibited synergistic, additive, and antagonistic effects when combined with levofloxacin, tigecycline, and daptomycin, respectively. Therefore, the rational selection of combination antibiotics can restore the efficacy of conventional drugs against multidrug-resistant pathogens such as MRSA, thereby enhancing the practical application value of HBPL.

## 2 Results

### 2.1 Physicochemical characterization of HBPL

**Figure 2:**
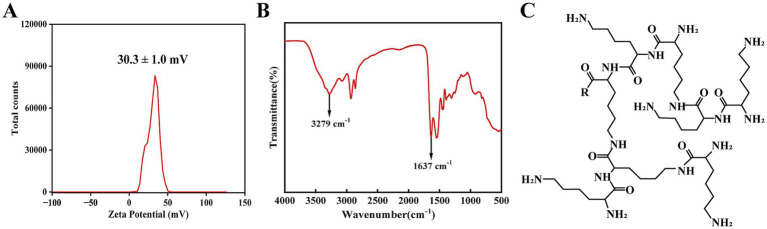
The physicochemical characterization of HBPL. **(A)** Zeta potential of HBPL (0.5 mg/mL) at pH 7 in water. This measurement was performed in triplicate, and the results are expressed as the mean ± standard deviation. **(B)** FT-IR image of HBPL (0.5 mg/mL). **(C)** Schematic diagram of the molecular structure of HBPL.

The fundamental properties of HBPL underpinning its interaction with bacterial cells were first characterized. Zeta potential analysis of a 0.5 mg/mL aqueous dispersion of HBPL revealed a net positive surface charge of 30.3 ± 1.0 mV at pH 7 (Figure 2A). This positive potential confirms the cationic nature of the polymer at bacteric physiological pH, which is essential for its initial electrostatic attraction to the negatively charged bacterial surface. Fourier-transform infrared (FT-IR) spectroscopy was used to confirm the chemical structure. The resulting spectrum displayed two characteristic absorption bands: a broad stretching band centered at 3,279 cm^−1^, corresponding to N–H vibrations, and a strong peak at approximately 1,637 cm^−1^, attributed to the amide I band (C=O stretch) and N–H bending vibrations (Figure 2B). These spectral features are consistent with the molecular structure of poly-L-lysine, confirming the abundance of amine and amide functionalities that define its chemical identity and function (Figure 2C). The number-average molecular weight (Mn) and weight-average molecular weight (Mw) of HBPL were 3,114 and 4,532, respectively. The poly dispersity index (PDI) value of HBPL is 1.46, which indicates a wide molecular weight distribution and molecular weight differences. HBPL exhibits a branching degree of 0.48, indicating a highly branched structure, which contributes to its excellent water solubility.

### 2.2 HBPL exhibits bactericidal activity against MRSA

**Figure 3:**
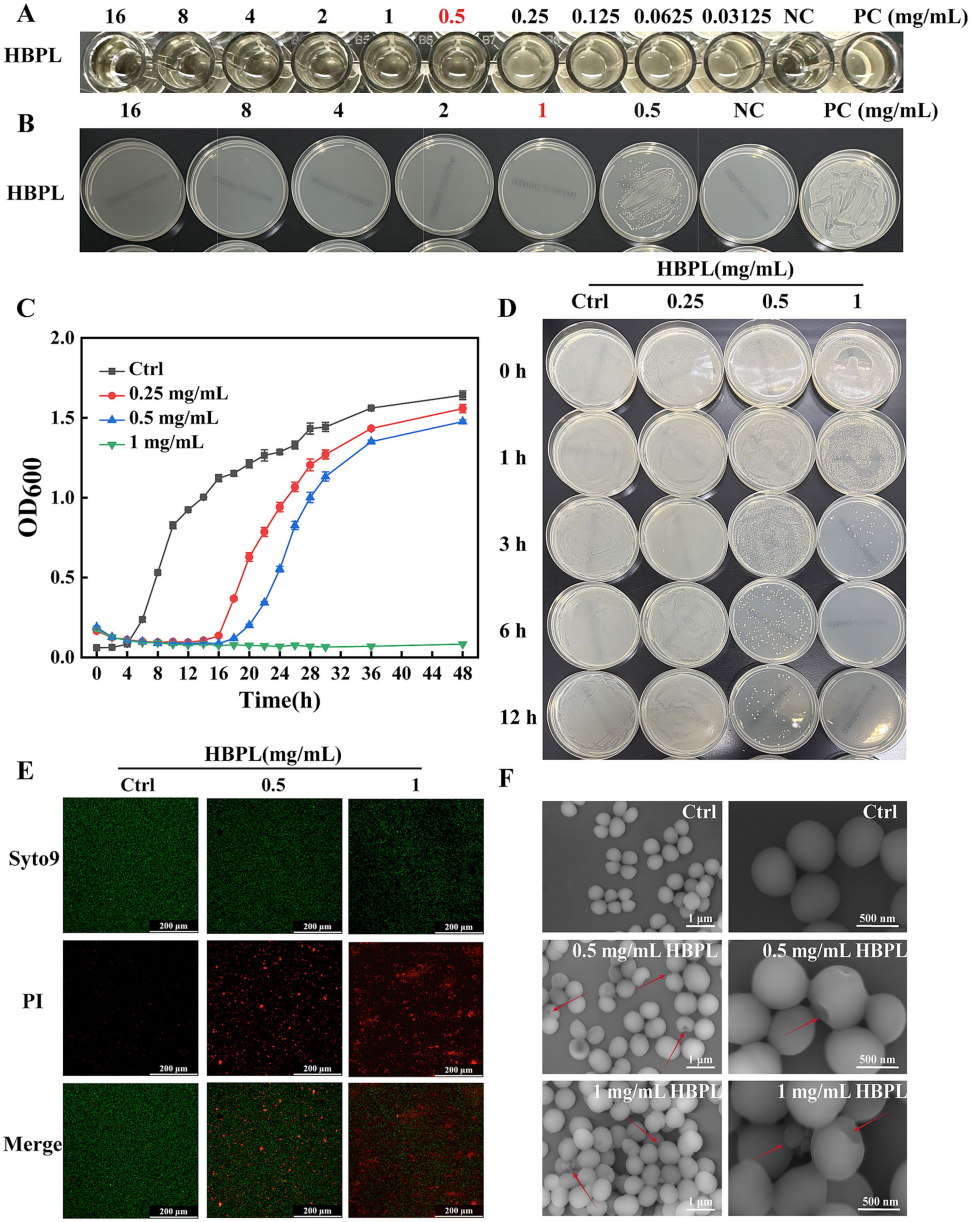
Antibacterial and bactericidal effects of HBPL on MRSA1. **(A)** The minimum inhibitory concentration of HBPL against MRSA1. **(B)** The minimum bactericidal concentration of HBPL for MRSA1. **(C)** Curve graph of the effect of HBPL on the growth of MRSA1. **(D)** The time and concentration dependence of HBPL on the killing of MRSA1. Images of MRSA1 treated with different concentrations of HBPL under fluorescence microscopy **(E)** and scanning electron microscopy **(F)**. Each experiment was in three technical replicates. (NC, negative control; PC, positive control).

The *in vitro* antibacterial efficacy of HBPL was evaluated against three MRSA strains, including the standard strain ATCC 43300 (MRSA 1) and two clinical isolates (MRSA 2, MRSA 3). As summarized in Table 1, HBPL demonstrated consistent and potent activity across all strains. The MIC, determined by the microbroth dilution method, was found to be 0.5 mg/mL for all three strains (Figure 3A; Supplementary Figure 1). The MBC, defined as the lowest concentration causing a ≥ 99.9% reduction in the initial inoculum, was 1.0 mg/mL (Figure 3B; Supplementary Figure 2). The low MBC/MIC ratio of 2 indicates that HBPL is a bactericidal agent rather than bacteriostatic agent.

**Table 1:** *In vitro* antibacterial activity of HBPL against MRSA.

Bacterial strain	MIC (mg/mL)	MBC (mg/mL)	Source
MRSA1	0.5	1.0	ATCC 43300
MRSA2	0.5	1.0	Clinical isolate
MRSA3	0.5	1.0	Clinical isolate

The dynamics of this bactericidal activities were further explored through growth curve and time-kill kinetic assays. Growth curve analysis showed that HBPL exerted a dose-dependent inhibitory effect on MRSA proliferation (Figure 3C, Supplementary Figure 3). In the presence of HBPL at its 0.5 × MIC (0.25 mg/mL), MIC (0.5 mg/mL) and 2 × MIC (1.0 mg/mL), the bacterial lag phase was significantly extended compared to the untreated control. At 2 × MIC, no significant increase in optical density was observed over a 48-h incubation period, indicating growth suppression. Time-kill kinetic assays confirmed the rapid nature of HBPL’s action (Figure 3D; Supplementary Figure 4). At a concentration of 2 × MIC (1.0 mg/mL), HBPL achieved a complete eradication (>5-log reduction) of an initial inoculum of 5 × 10^5^ CFU/mL within just 6 h. Even at its MIC, HBPL induced significant bacterial killing within 12 h, demonstrating a potent and time-dependent bactericidal effect.

### 2.3 HBPL induces irreversible damage to the MRSA cell envelope

To visualize the mechanism of action at the cellular level, confocal laser scanning microscopy (CLSM) and scanning electron microscopy (SEM) were employed. CLSM with dual fluorescent staining (SYTO 9 for live cells with intact membranes, and propidium iodide [PI] for dead cells with compromised membranes) provided direct evidence of membrane disruption (Figure 3E; Supplementary Figure 5). Untreated control MRSA cells fluoresced brightly green, indicating intact membranes. In contrast, after a 6-h treatment with HBPL at MIC and 2 × MIC, a dose-dependent shift in fluorescence was observed. The population of green cells diminished while red-fluorescing cells became predominant, disruption of membrane integrity that allowed PI to enter and stain the bacterial nucleic acids.

SEM provided high-resolution morphological details of this damage (Figure 3F; Supplementary Figure 6). Control MRSA cells appeared as uniform, smooth-surfaced spheres, typical of healthy staphylococci. Treatment with HBPL at 0.5 mg/mL induced visible surface damage, including pitting and depressions, and caused the cells to aggregate. At 1.0 mg/mL, more damage was observed, characterized by cell-wall invagination, membrane collapse, and the formation of cellular debris. These images provide unequivocal visual evidence that HBPL’s primary mode of action involves the physical destruction of the bacterial cell envelope.

### 2.4 HBPL disrupts biofilm formation and affects cellular protein profiles

**Figure 4:**
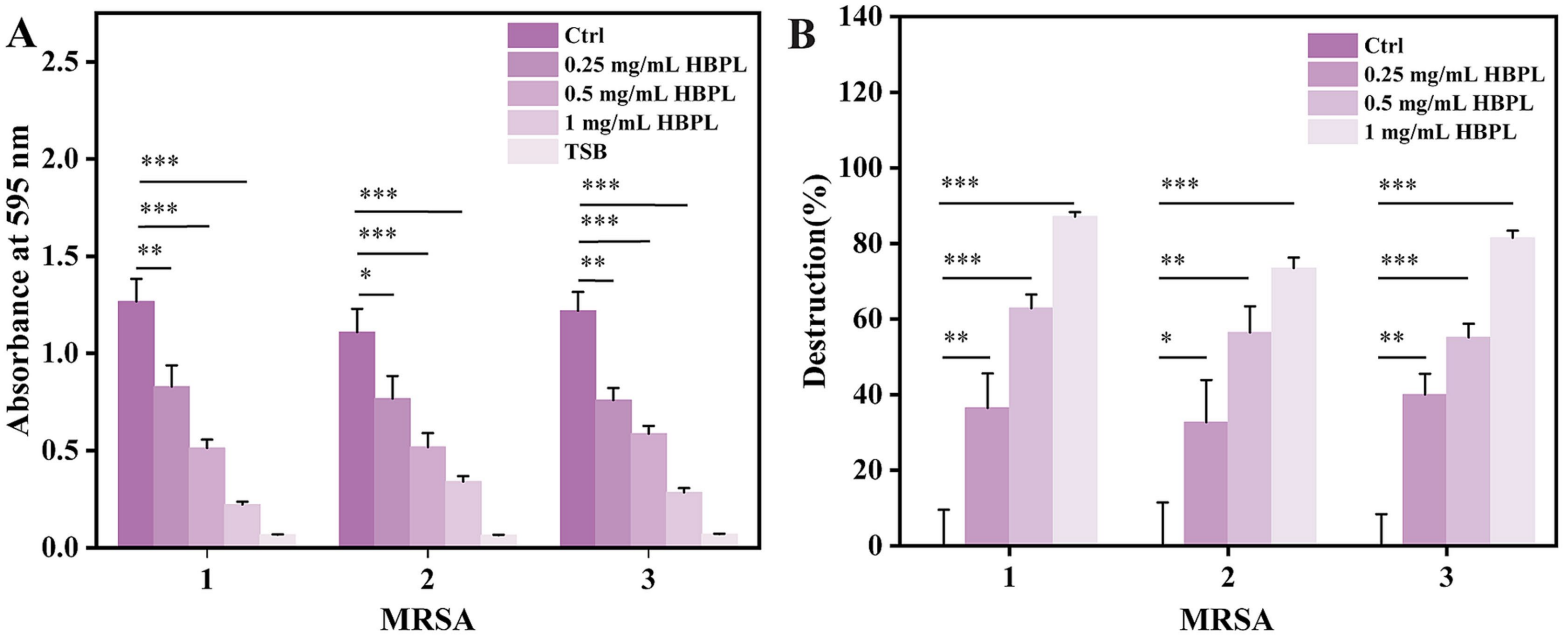
Anti-biofilm activity of HBPL against three strains of MRSA. Absorbance at 595 nm **(A)** and destruction ratio **(B)** for each group. Each experiment was repeated three times independently and included three biological replicates. Error bars represent standard error of the mean. ^*^p < 0.05, ^**^p < 0.01, and ^***^p < 0.001.

Given the critical role of biofilms in MRSA pathogenesis, the ability of HBPL to inhibit their formation was quantified using a crystal violet staining assay (Figure 4A). In the experiment, MRSA strains were pre-cultured in a microplate for 12 h to allow for the establishment of a preliminary biofilm. Subsequently, the cultures were treated, respectively, with 0, 0.25, 0.5 and 1.0 mg/mL HBPL for 36 h. The results demonstrated a dose-dependent anti-biofilm effect of HBPL. At concentrations of 0.25 mg/mL (0.5 × MIC) and 0.5 mg/mL (MIC), HBPL significantly reduced biofilm biomass compared to the untreated control. At the highest concentration of 1.0 mg/mL (2 × MIC), HBPL inhibited biofilm formation by 87, 73, and 81% for the three MRSA strains, respectively (Figure 4B). This indicates that HBPL can interfere with the initial stages of biofilm development.

To assess the broader downstream effects of HBPL treatment on cellular metabolism, total protein extracts from treated and untreated MRSA1 were analyzed by SDS-PAGE (Supplementary Figure 7). The protein profile of untreated control cells showed intensely stained bands. Following treatment with increasing concentrations of HBPL, a clear dose-dependent effect was observed: the protein bands became progressively fainter and narrower, with some disappearing entirely at higher concentrations. This global reduction in detectable protein suggests that the membrane disruption caused by HBPL leads to widespread metabolic collapse, either through the leakage of cytoplasmic proteins, the halting of protein synthesis, or subsequent protein degradation.

### 2.5 Prolonged exposure to HBPL induces stable, low-level resistance

**Figure 5:**
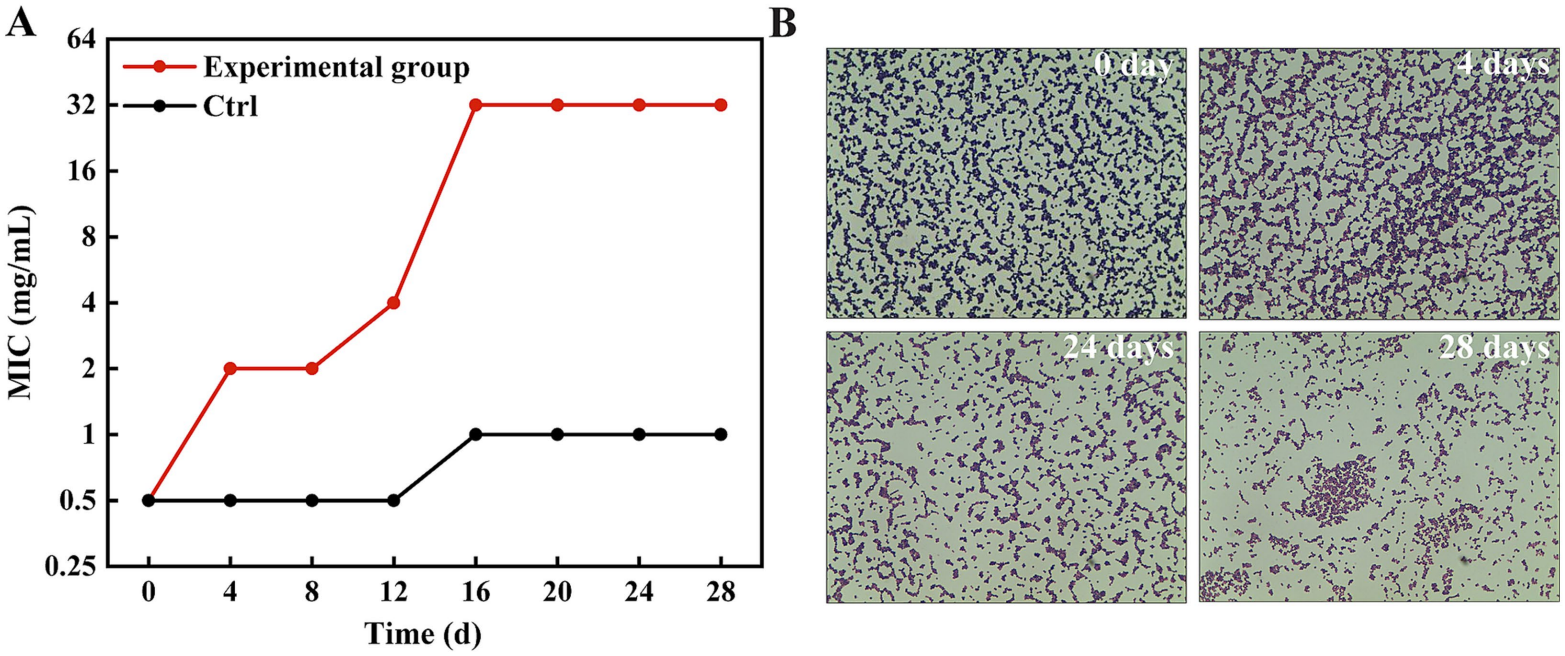
MRSA1 develops resistance after being induced by concentration gradient HBPL. **(A)** MIC values of 28 passages under co-culture of HBPL and MRSA 1 with different concentration gradients. Each experiment was in three technical replicates. **(B)** MRSA1 after induction with different concentration gradients of HBPL (Gram staining, ×1,000).

To investigate the potential for resistance development, a critical consideration for any new antimicrobial, MRSA1 was subjected to serial passage in the presence of escalating concentrations of HBPL for 24 passages. The MIC value progressively increased, rising four-fold within the first four passages and eventually stabilizing at a 64-fold increase compared to the initial MIC of the parent strain (Figure 5A). The MIC of MRSA 1 in the absence of HBPL increased by only one dilution gradient. To assess the stability of this acquired phenotype, the adapted strain was subsequently passaged four times in drug-free medium. The elevated MIC did not revert to the baseline level, indicating that the induced resistance was a stable trait.

Gram staining of the adapted strains revealed striking morphological and physiological changes (Figure 5B). While the parent strain exhibited typical Gram-positive characteristics (purple staining, uniform cocci), the HBPL-adapted strains showed a partial shift toward Gram-negative-like staining (pink/red cells) and a noticeable increase in cell size. These observations suggest that the mechanism of reduced susceptibility involves structural remodeling of the bacterial cell envelope.

To assess the fitness cost of this adaptation, we performed comparative growth curve analysis of the parent strain and the HBPL-adapted strain in HBPL-free medium (Supplementary Figure 8). The adapted strain exhibited a reduced maximum growth rate. This demonstrates a fitness defect associated with the acquired resistance.

### 2.6 HBPL displays drug-specific synergistic and antagonistic interactions in combination therapies

The potential of HBPL to act as an adjuvant to conventional antibiotics was preliminarily evaluated using checkerboard assays against three MRSA strains. FICI was calculated to classify the interactions as synergistic, additive, indifferent, or antagonistic. The results, summarized in Table 2, indicates specific, mechanism-dependent interactions.

**Table 2:** Fractional inhibitory concentration index of HBPL in combination with antibiotics against MRSA.

Bacteria	Drug A	MIC_A_ (μg/mL)	MIC_AB_ (μg/mL)	Drug B	MIC_B_ (mg/mL)	MIC_BA_ (mg/mL)	FICI	Interpretation
MRSA1	Levofloxacin	0.25	0.064	HBPL	0.5	0.125	0.500	Synergy
MRSA2	Levofloxacin	16	0.25	HBPL	0.5	0.125	0.266	Synergy
MRSA3	Levofloxacin	64	1	HBPL	0.5	0.125	0.266	Synergy
MRSA1	Tigecycline	0.125	0.032	HBPL	0.5	0.25	0.756	Additive
MRSA2	Tigecycline	0.125	0.016	HBPL	0.5	0.25	0.628	Additive
MRSA3	Tigecycline	0.125	0.032	HBPL	0.5	0.25	0.756	Additive
MRSA1	Daptomycin	1	2	HBPL	0.5	0.064	2.215	No interaction
MRSA2	Daptomycin	1	4	HBPL	0.5	0.064	4.125	Antagonism
MRSA3	Daptomycin	1	2	HBPL	0.5	0.25	2.500	No interaction

**Figure 6:**
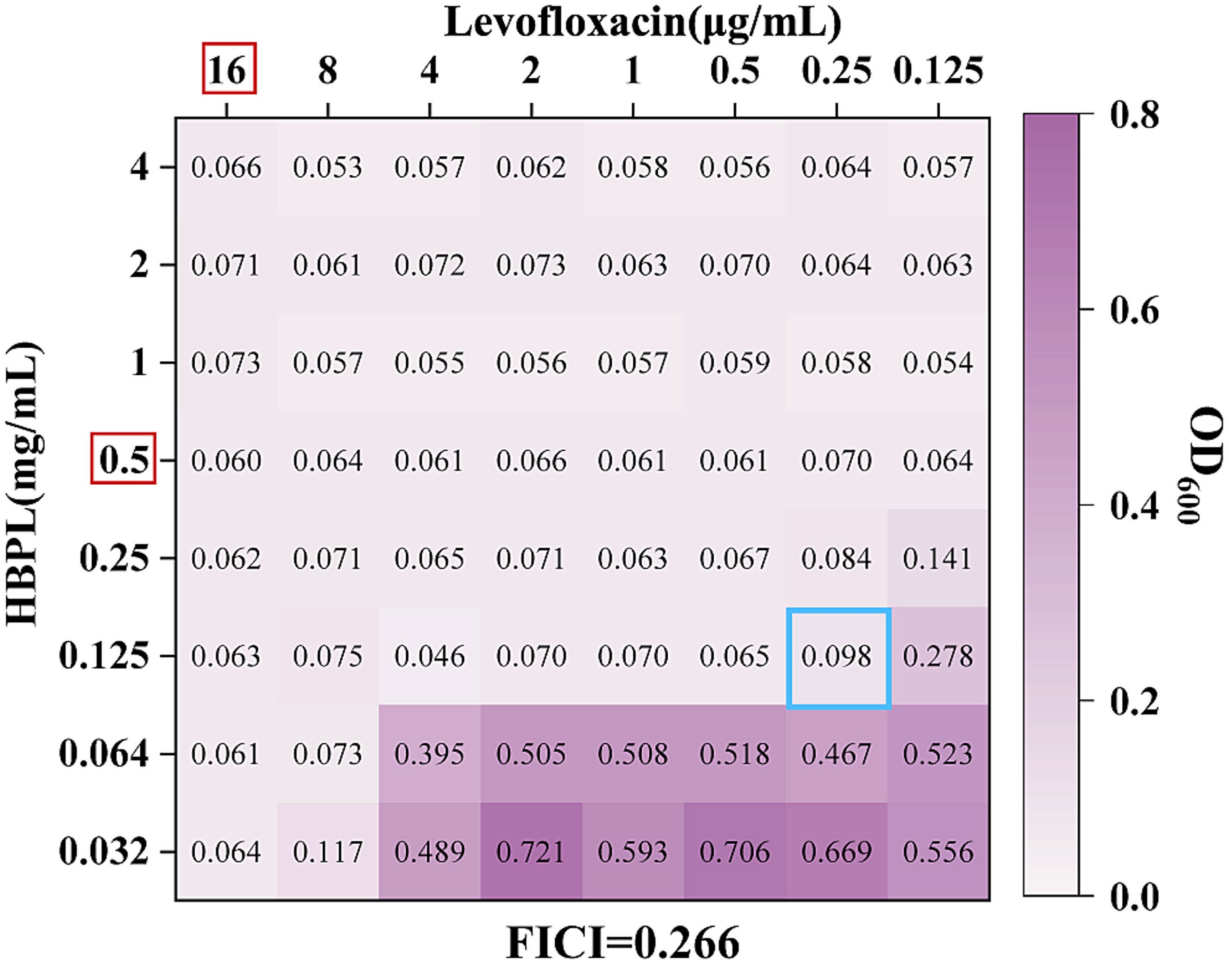
The effect of the combination of HBPL and levofloxacin to MRSA 2. The red box indicates the MIC of the single drug against MRSA2. The hole marked in the blue box is the FICI calculation hole.

Synergy (FICI ≤ 0.5): A consistent and potent synergistic effect was observed when HBPL was combined with the fluoroquinolone levofloxacin. The FICI values were 0.500, 0.266 (Figure 6), and 0.266 for the three strains, indicating that the combination may be more effective than either agent alone, pointing toward a potential synergistic interaction.

As shown in Figure 6, an OD_600_ nm value greater than 0.1 indicates bacterial growth. The red box highlights the MIC of the single drug against MRSA2. We observed that the MIC of the HBPL combination was 0.125 mg/mL, while the MIC of the levofloxacin combination was 0.25 μg/mL. The blue box marked the FICI calculation hole, and the FICI value of this hole was 0.266, which is less than 0.5, indicating a synergistic interaction between levofloxacin and HBPL.

Additive effect (0.5 < FICI ≤ 1.0): An additive interaction was found with tigecycline, a glycylcycline (FICI values: 0.756, 0.628, 0.756). This suggests the combined effect is roughly equal to the sum of their individual effects.

No interaction (1.0 < FICI ≤ 4.0) and antagonism (FICI > 4.0): The combination of HBPL with daptomycin, a cyclic lipopeptide, resulted in no interaction for MRSA1 and MRSA3 (FICI: 2.125, 2.500) and clear antagonism for MRSA2 (FICI: 4.125). This indicates that the combination is less effective than the individual agents.

These results indicates that the outcome of combining HBPL with an antibiotic is not arbitrary but likely to be by the specific mechanisms of action of the partner drugs.

## 3 Discussion

This study provides a comprehensive mechanistic evaluation of HBPL as an anti-MRSA agent, revealing a multi-modal mechanism of action, potent anti-biofilm activity, and specific interactions when combined with conventional antibiotics. The findings not only show HBPL as a promising antimicrobial polymer but also offer insights into the rational design of combination therapies against multidrug-resistant pathogens.

The collective evidence from this study converges to support a model of rapid, physically-mediated bactericidal activity. Unlike conventional antibiotics that target specific enzymes or metabolic pathways, HBPL’s action is a direct assault on the structural integrity of the MRSA cell envelope. The process initiates with the electrostatic attraction between the cationic HBPL and the net-negative surface of the *S. aureus* cell wall. In Gram-positive bacteria, this negative charge is primarily conferred by the abundant phosphate groups within wall teichoic acids (WTAs), which are anionic polymers covalently linked to the peptidoglycan (Campbell et al., 2012). This charge-based “docking” is the crucial first step that concentrates the polymer at the bacterial surface. Following adsorption, HBPL penetrates and disrupts the cytoplasmic membrane. This is unequivocally demonstrated by the CLSM results, where the influx of the membrane-impermeable dye PI indicates membrane damage. The mechanism is analogous to that of other membrane-active agents, where amphipathic molecules insert into the lipid bilayer, disrupt its organization, and lead to the formation of pores or transient defects (Shai, 2002). The high-resolution SEM images, showing surface pitting, invagination, and eventual cellular collapse, provide morphological corroboration of this destructive process.

The loss of membrane integrity has immediate and fatal consequences. It leads to the dissipation of the proton motive force, leakage of essential ions and small metabolites, and an inability to maintain cellular homeostasis (Hurdle et al., 2011). This explains the rapid, concentration-dependent killing observed in the time-kill assays. The global reduction in cellular protein bands seen on SDS-PAGE is a downstream consequence of this collapse, reflecting a combination of protein leakage from the compromised cell, halted protein synthesis due to energy depletion, and potential degradation by released proteases. This multi-pronged physical attack provides few avenues for the bacteria to mount a successful defense, explaining its bactericidal effect.

The ability of HBPL to inhibit biofilm formation of three MRSA strains by 87, 73, and 81%, respectively, at a concentration of 1.0 mg/mL is a key finding with therapeutic implications. This potent activity likely stems from a dual mechanism. The primary mode of action is the prevention of the initial attachment phase. Biofilms begin when planktonic bacteria adhere to a surface (Vanamala et al., 2021). As demonstrated by the time-kill assays, HBPL rapidly eradicates these free-floating cells, effectively neutralizing the building blocks of the biofilm before it can be established. Secondly, cationic polymers can directly interfere with the integrity of the EPS matrix. The matrix is held together by various components, including negatively charged eDNA (Goodman and Bakaletz, 2022). The cationic nature of HBPL could allow it to bind to and disrupt this eDNA scaffold, destabilizing the biofilm structure. Furthermore, the membrane disruption caused by HBPL could interfere with bacterial communication systems like quorum sensing, which are essential for coordinating biofilm maturation (Rémy et al., 2018).

The observations that after continuous concentration gradient treatment with HBPL, the MIC of MRSA1 increases 64-fold, whereas in the absence of HBPL, the MIC of MRSA1 only increases by one dilution. This is an important finding that warrants further explanation. This does not represent the acquisition of resistance via a single target-site mutation, as is common for many conventional antibiotics. Instead, it reflects a complex, adaptive stress response involving a major overhaul of the cell envelope. The striking morphological changes observed—a shift toward Gram-negative-like staining and cell swelling—are phenotypic evidence of this remodeling. To assess the fitness cost of this adaptation, we performed comparative growth curve analysis of the parent strain and the HBPL-adapted strain in HBPL-free medium. The adapted strain exhibited a reduced maximum growth rate, indicating a fitness defect associated with the acquired resistance. This fitness cost may limit the persistence and spread of such resistant variants in the absence of continuous selective pressure from HBPL. A plausible molecular mechanism for this adaptation involves the modification of the cell surface charge to reduce electrostatic attraction to the cationic polymer. Bacteria have evolved sophisticated systems to resist cationic AMPs, including the D-alanylation of teichoic acids (mediated by the *dlt* operon) and the lysinylation of membrane phospholipids (mediated by the *mprF* gene) (Tajer et al., 2024). While these are well-documented pathways for AMP resistance, the specific genetic and molecular basis for HBPL resistance in our adapted MRSA strain remains to be elucidated. Our current interpretation is therefore largely speculative and based on phenotypic evidence. Future studies employing genomic sequencing, transcriptomic analysis, and targeted gene knockout experiments are essential to definitively identify the molecular mechanisms underlying this adaptive resistance.

The results of the checkerboard method indicate that the success of combination therapy of HBPL with antibiotics is not fortuitous but is governed by underlying mechanistic principles (Acar, 2000). This success is likely due to the specific mechanisms of the cooperating drugs. Different joint susceptibility results highlight the potential of HBPL as an adjuvant, but also warn against misuse. We compared the mechanisms of action of three antibiotics: levofloxacin, which belongs to the fluoroquinolone class, primarily targets DNA gyrase and topoisomerase IV in the cytoplasm, inhibiting DNA replication and separation, leading to DNA damage and cell death (Maddiboyina et al., 2023); tigecycline, belonging to the glycylcycline class, targets the 30S ribosomal subunit in the cytoplasm, inhibiting protein synthesis by preventing the entry of aminoacyl-tRNA (Maddiboyina et al., 2023); daptomycin is a cyclic lipopeptide antibiotic that acts on the cell membrane in a calcium-dependent manner, leading to rapid depolarization and cell death (Kotsogianni et al., 2021).

The potent synergy (FICI ≤ 0.5) between HBPL and levofloxacin is best explained by a “two-hit” cooperative model (Ng et al., 2013). HBPL acts as the first hit, functioning as a permeabilizing agent that disrupts the primary barrier—the cell membrane. This disruption creates entry points for levofloxacin, the second hit, allowing it to flood the cytoplasm and reach its intracellular targets, DNA gyrase and topoisomerase IV, at concentrations that would otherwise be ineffective (Qandeel et al., 2025). This mechanism, where a membrane-active agent facilitates the uptake of an intracellular-targeting antibiotic, is a classic and powerful strategy for overcoming resistance and restoring the efficacy of established drugs (Acar, 2000).

The additive effects are also mechanistically logical. The additive effect with the protein synthesis inhibitor tigecycline suggests that while HBPL-mediated membrane disruption may offer some modest benefit for drug entry, it does not dramatically potentiate its action in the way it does for levofloxacin, possibly due to differences in drug structure, charge, or transport kinetics (Korczak et al., 2024).

The observed antagonism (FICI > 4) between HBPL and daptomycin is perhaps the most meaningful finding. We propose a mechanism of competitive antagonism at the membrane level. Both HBPL and daptomycin are cationic, membrane-active agents that target the bacterial cell membrane. Their efficacy relies on binding to anionic phospholipid domains within the membrane to initiate disruption (Hurdle et al., 2011). When used in combination, these two molecules likely compete for the same limited binding sites on the staphylococcal membrane. This competition could lead to steric hindrance, preventing either agent from achieving the optimal concentration and orientation required for efficient membrane disruption, thereby reducing their combined efficacy below that of either agent used alone (Kleinman, 2024). This result serves as a potential cautionary tale in combination therapy design: combining agents with identical or overlapping sites of action can be counterproductive.

Perhaps one of the most significant, yet unexplored, implications of HBPL’s mechanism relates to bacterial persister cells. These dormant cells are a major cause of chronic infection relapse because they are tolerant to conventional antibiotics that target active metabolic processes (Kim et al., 2018). However, agents that act via physical membrane disruption, like HBPL, should theoretically be effective against persisters because even dormant cells must maintain their membrane integrity to survive (Hurdle et al., 2011). This positions HBPL not merely as an alternative to antibiotics but as a potential solution to one of the most intractable challenges in infectious disease.

This study is, however, subject to the limitations inherent to *in vitro* research. The experiments were conducted in idealized culture conditions, lacking the complexities of the host environment, such as immune cells and host proteins. Therefore, future work must focus on translating these promising findings. Key next steps should include: (1) comprehensive *in vivo* efficacy studies in established animal models of MRSA infection (e.g., skin infection, bacteremia); (2) rigorous toxicological profiling to determine the therapeutic window and assess biocompatibility in a physiological context (Deepak et al., 2024); and (3) pharmacokinetic and pharmacodynamic (PK/PD) studies to understand the absorption, distribution, metabolism, and excretion of HBPL. These investigations are essential to fully realize the clinical potential of HBPL in the post-antibiotic era.

## 4 Conclusion

In conclusion, this study indicates HBPL as a multi-functional antimicrobial polymer with potential for combating infections caused by MRSA. Its primary mechanism of action is a rapid, physical disruption of the bacterial cell envelope, which is less susceptible to conventional resistance development. This membrane-active mechanism not only leads to efficient killing of planktonic MRSA but also translates into a ability to inhibit the formation of resilient bacterial biofilms. Crucially, this work provides a mechanistic blueprint for the rational design of combination therapies. The synergy observed between HBPL and the intracellular-targeting antibiotic levofloxacin indicates the strategy of using membrane-permeabilizing polymers to restore the efficacy of existing drugs. Conversely, the antagonism with daptomycin highlights the critical importance of avoiding combinations of agents with competing mechanisms of action. While the potential for adaptive resistance development necessitates cautious and strategic application, the overall data support the continued development of HBPL as a valuable therapeutic agent or adjuvant. Its unique physical mechanism suggests it may also be effective against dormant persister cells, offering a potential strategy to address the challenge of chronic, relapsing infections. Further *in vivo* investigation is warranted to translate these promising *in vitro* findings into tangible clinical solutions for the post-antibiotic era.

## 5 Materials and methods

### 5.1 Materials and reagents

Hyper-branched poly-L-lysine (HBPL) was provided by Bohui (Zhejiang) Biotechnology Co., Ltd. Crystal violet, Tryptic soy broth with glucose supplementation (TSB) and Luria-Bertani (LB) broth were purchased from Sangon Biotech (Shanghai, China). The One-Step PAGE Gel Fast Preparation Kit (12%) was obtained from Vazyme Biotech Co., Ltd. (Nanjing, China). Levofloxacin, daptomycin, and tigecycline were supplied by Wenzhou Kangtai Biological Technology Co., Ltd. (Wenzhou, China). All other reagents were of analytical grade.

### 5.2 Physicochemical characterization of HBPL

The zeta potential of HBPL was determined using a Malvern Zetasizer 90 (Malvern Instruments, United Kingdom). For Fourier-transform infrared (FT-IR) spectroscopy, a 0.5 mg/mL solution of HBPL was dried to form a thin film. Spectra were acquired on a Nicolet iS50 spectrometer (Thermo Fisher Scientific, United States) to analyze the molecular structure and chemical composition. The other characteristic parameters of HBPL were provided by Bohui (Zhejiang) Biotechnology Co., Ltd.

### 5.3 Bacterial strains and culture conditions

Three MRSA strains were used: the standard reference strain ATCC 43300 (MRSA1) and two clinical isolates (MRSA2, MRSA3) obtained from patient samples. All strains were preserved at −80 °C in the laboratory’s collection. Identity was confirmed using Matrix-Assisted Laser Desorption/Ionization Time-of-Flight Mass Spectrometry (MALDI-TOF MS) and the VITEK 2 Compact system. For experiments, strains were recovered on LB agar, inoculated into LB broth, and incubated overnight at 35 °C with shaking at 150 rpm to reach the logarithmic growth phase. The bacterial suspension was then washed with sterile phosphate-buffered saline (PBS) and adjusted to a turbidity of 0.5 McFarland units.

### 5.4 Determination of MIC and MBC

MIC and MBC were determined using the broth microdilution method following CLSI guidelines (Sun et al., 2025). A stock solution of HBPL was serially diluted two-fold in a 96-well microtiter plate with LB broth, starting from a concentration of 16 mg/mL. An equal volume of bacterial suspension (final concentration 5 × 10^5^ CFU/mL) was added to each well (final volume 100 μL). Plates were incubated at 35 °C for 18–24 h. The MIC was defined as the lowest concentration of HBPL with no visible bacterial growth. To determine the MBC, 100 μL aliquots from all clear wells were plated onto LB agar and incubated at 35 °C for 18–24 h. The MBC was defined as the lowest concentration that resulted in no colony formation.

### 5.5 Growth curve analysis

Bacterial suspensions were diluted in LB broth to 5 × 10^5^ CFU/mL. HBPL was added to final concentrations of 0.5 × MIC, 1 × MIC, and 2 × MIC. A culture without HBPL served as the control. The cultures were incubated at 35 °C with shaking at 150 rpm. The optical density at 600 nm (OD_600_) was measured at specified time points over 48 h using a SpectraMax Plus microplate reader.

### 5.6 Time-kill kinetic assay

Bacterial suspensions were prepared at 5 × 10^5^ CFU/mL in LB broth and treated with HBPL at 0.5 × MIC, 1 × MIC, and 2 × MIC. At time points of 0, 1, 3, 6, and 12 h, 200 μL aliquots were removed, and plated on LB agar. Plates were incubated at 35 °C for 18–24 h, after which colonies were counted to determine the number of viable bacteria (CFU/mL).

### 5.7 Confocal laser scanning microscopy

Bacterial suspensions were diluted in LB broth to 10^8^ CFU/mL. MRSA cells were treated with HBPL at MIC and 2 × MIC for 6 h at 35 °C. Untreated cells served as a control. The bacteria were collected by centrifugation, washed with PBS, and stained with a mixture of SYTO 9 and PI dyes for 10 min in the dark. After washing, the cells were mounted on a glass slide and observed using a Leica Stellaris 5 confocal microscope at 20× magnification to visualize membrane integrity.

### 5.8 Scanning electron microscopy

Bacterial suspensions were diluted in LB broth to 10^8^ CFU/mL. MRSA cells were treated with HBPL at MIC and 2 × MIC for 6 h at 35 °C. Untreated cells served as a control. The bacteria were collected by centrifugation, washed with PBS, the cells were fixed with 2.5% glutaraldehyde in PBS overnight at 4 °C. The fixed cells were washed with PBS and dehydrated through a graded ethanol series (30 to 100%). The dehydrated samples were dropped onto silicon wafers, air-dried, sputter-coated with gold, and imaged using a Hitachi SU8600 field-emission SEM at 20× and 60× magnification.

### 5.9 SDS-PAGE analysis of cellular proteins

MRSA 1 was diluted in LB broth to 10^8^ CFU/mL. Then, treated with HBPL at 0.5 × MIC, 1 × MIC, and 2 × MIC for 6 h. Cells were harvested, washed, and lysed using lysozyme followed by a RIPA lysis buffer containing a protease inhibitor (PMSF) and a nuclease. The total protein concentration was determined, and equal amounts of protein lysate were mixed with 5 × loading buffer, boiled, and resolved on a 12% SDS-polyacrylamide gel. The gel was stained with Coomassie brilliant blue to visualize the protein bands.

### 5.10 Biofilm inhibition assay

Bacterial suspensions were diluted in LB broth to 5 × 10^5^ CFU/mL. MRSA suspensions were added to TBSg medium and incubated at 35 °C for 12 h. The suspensions were then treated with 0.5 × MIC, MIC and 2 × MIC concentrations of HBPL, with no HBPL as the control. After incubation at 35 °C for 36 h, the planktonic cells were removed, and the wells were washed with PBS. The remaining biofilms were fixed with methanol and stained with 0.1% crystal violet. The dye was dissolved in 95% ethanol, and the absorbance was measured at 595 nm to quantify the biofilm biomass. Each experiment was repeated three times independently and included three biological replicates. Statistical analysis and data analysis was performed using SPSS Version 21.0 (IBM Corp., Armonk, NY, United States). The difference in biofilm formation was analyzed using a *t*-test. Error bars represent standard error of the mean. ^*^p < 0.05, ^**^p < 0.01, and ^***^p < 0.001.

### 5.11 *In vitro* resistance induction study

To investigate the possibility of resistance development, we used elevated MIC values to reflect the development of resistance of MRSA to HBPL (Sun et al., 2025). The experiment selected MRSA 1 as the study object. Bacteria were co-cultured with HBPL at concentrations of 0.5 × MIC, MIC, 2 × MIC, 4 × MIC, 8 × MIC, and 16 × MIC. The MIC of the adaptive strains was measured after each co-culture concentration. The specific process involved using a 0.5 McFarland standard turbidity bacterial suspension (approximately 1.5 × 10^8^ CFU/mL) of 50 μL added to 9,950 μL of LB medium, exposed to 0.5 × MIC of HBPL for co-culture at 35 °C for 18–24 h, centrifuged, and washed three times with PBS. The adaptive strain was then used at a 0.5 McFarland standard turbidity bacterial suspension (approximately 1.5 × 10^8^ CFU/mL), with 50 μL of the suspension added to 9,950 μL of LB medium and exposed to 0.5 × MIC of HBPL for co-culture again. This process was repeated four times, and the MIC of the adaptive strain at this exposure concentration was measured. Subsequently, the adaptive strain was exposed to higher concentrations of HBPL for co-culture, and this cycle was repeated six times. After 24 generations, the adaptive strain was subcultured in HBPL-free medium for four generations to stabilize the phenotype. Additionally, the original MRSA 1 strain was continuously subcultured in LB broth for 28 generations without adding HBPL as a spontaneous mutation control, and its MIC was also measured. Daily HBPL concentration in each group was shown in Supplementary Table 1. Each experiment was in three technical replicates. All MIC values were recorded. Gram staining was performed on both the original and adaptive strains to observe morphological changes. Meanwhile, we conducted growth analysis of the parental strain and HBPL-adapted strains in a medium without HBPL. The cultures were incubated at 35 °C with shaking at 150 rpm. The optical density at 600 nm was measured at specified time points over 48 h using a Spectra Max Plus microplate reader. Statistical analysis and data analysis was performed using SPSS Version 21.0 (IBM Corp., Armonk, NY, United States). The difference in optical density was analyzed using a *t*-test. Error bars represent standard error of the mean. ^*^p < 0.05, ^**^p < 0.01, and ^***^p < 0.001.

### 5.12 Checkerboard synergy assay

#### 5.12.1 Single-drug susceptibility testing (microdilution method)

After presetting the initial concentrations of three categories of antimicrobial agents (levofloxacin 128 μg/mL, tigecycline 32 μg/mL, daptomycin 32 μg/mL), a 2-fold dilution series was prepared in 96-well plates, with 50 μL of drug solution per well. Fifty microliters of bacterial suspension (final concentration 5 × 10^5^ CFU/mL) was added to each well to complete the final volume of 100 μL. Each drug-bacterial combination was tested in triplicate. Negative controls were set using LB broth alone, and positive controls were set using bacterial suspension alone. The plates were incubated at 35 °C for 18–24 h, and results were observed.

#### 5.12.2 Combined drug susceptibility test (checkerboard method)

Assessing the antibacterial activity of HBPL combined with three categories of antimicrobial agents (levofloxacin, tigecycline, daptomycin) busing the checkerboard method (Sun et al., 2025). Refer to the single drug MIC, the 2-fold serial dilutions of each compound within an appropriate concentration range were mixed to form an 8 × 8 matrix in a 96-well plate. Levofloxacin, tigecycline, daptomycin were diluted horizontally, while HBPL was diluted vertically. Bacterial suspensions were finally diluted to 5 × 10^5^ CFU/mL (final volume 100 μL). After incubation at 35 °C for 18–24 h, the MIC in combination was read. Each drug-bacterial combination was tested in triplicate. The FICI was calculated using the formula:


$$\text{FICI}=\frac{{\mathrm{MIC}}_{\mathrm{AB}}}{{\mathrm{MIC}}_{A}}+\frac{{\mathrm{MIC}}_{\mathrm{BA}}}{{\mathrm{MIC}}_{B}}$$

MIC_A_: MIC_A_ alone; MIC_AB_: MIC_A_ combo and MIC_B_: MIC_B_ alone; MIC_BA_: MIC_B_ combo.

FICI ≤ 0.5 indicates synergism effect between the 2 drugs; 0.5 < FICI ≤ 1 indicates cumulative effect between the 2 drugs; 1 < FICI ≤ 4 indicates no interaction effect between the 2 drugs; FICI > 4 indicates antagonism effect between the 2 drugs (Liu et al., 2024).

## Data Availability

The raw data supporting the conclusions of this article will be made available by the authors, without undue reservation.
