# Supplementary material for: Antibacterial mechanism of hyper-branched poly-L-lysine against methicillin-resistant Staphylococcus aureus and its synergistic and antagonistic interactions with conventional antibiotics
Source: Front Microbiol. 2025 Oct 8;16:1676135. doi: 10.3389/fmicb.2025.1676135 (PMC12540308; doi:10.3389/fmicb.2025.1676135)
Supplement: Supplementary file 1 [file Data_Sheet_1.docx]

Supplementary Material

Antibacterial Mechanism of Hyper-branched Poly-L-lysine against Methicillin-Resistant Staphylococcus aureus and Its Synergistic and Antagonistic Interactions with Conventional Antibiotics


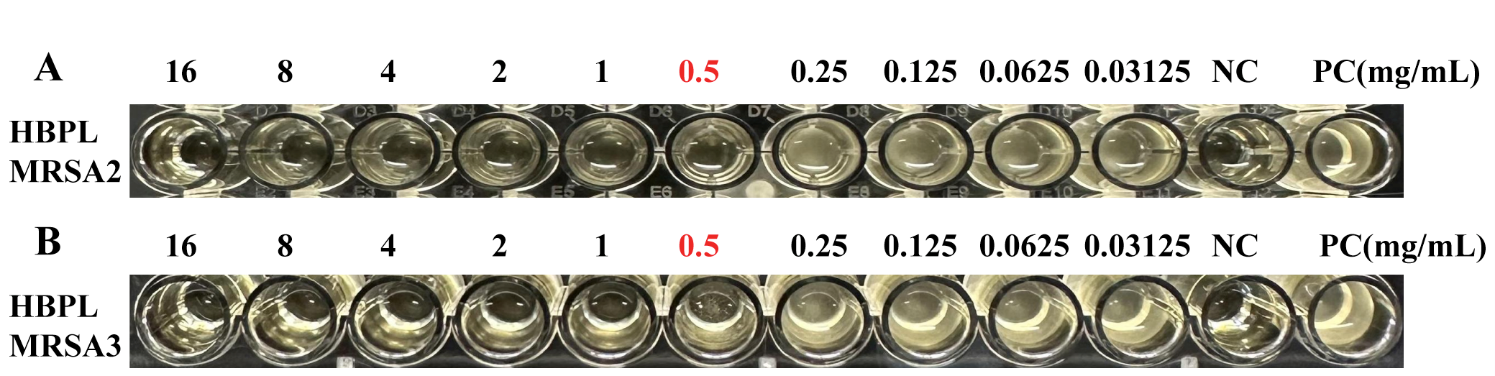


**Supplementary Figure 1.** (A) The minimum inhibitory concentration of HBPL against MRSA2 (A) and MRSA3 (B). (NC, Negative control; PC, Positive control)


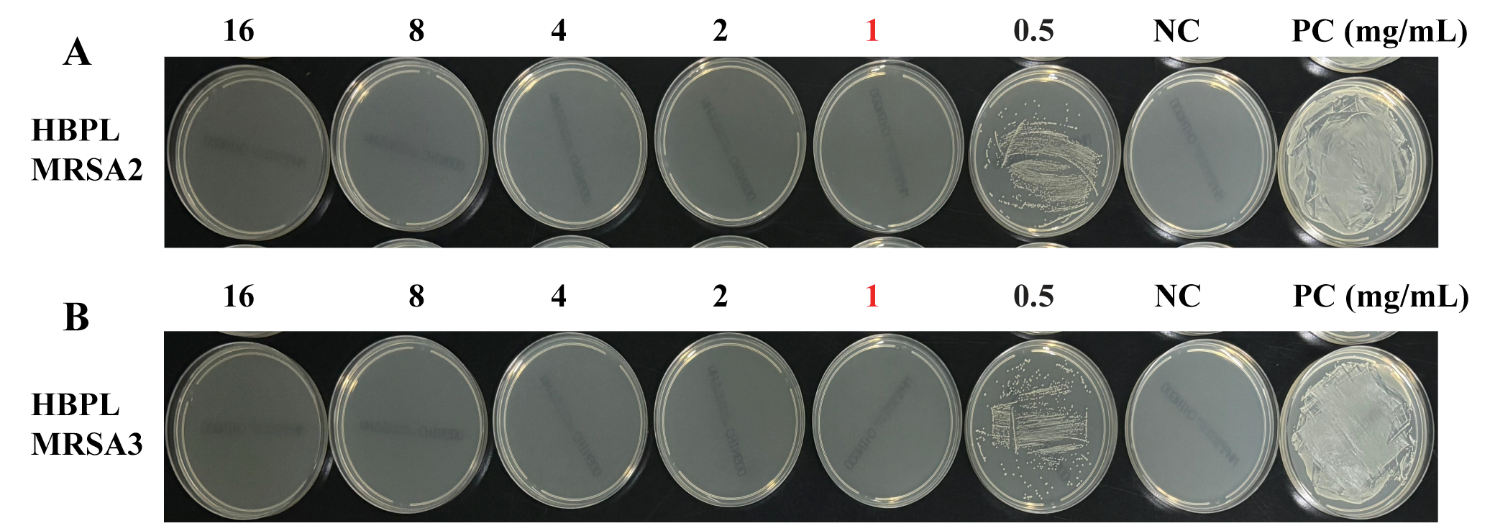


**Supplementary Figure 2.** The minimum bactericidal concentration of HBPL for MRSA2 (A) and MRSA3 (B). (NC, Negative control; PC, Positive control)


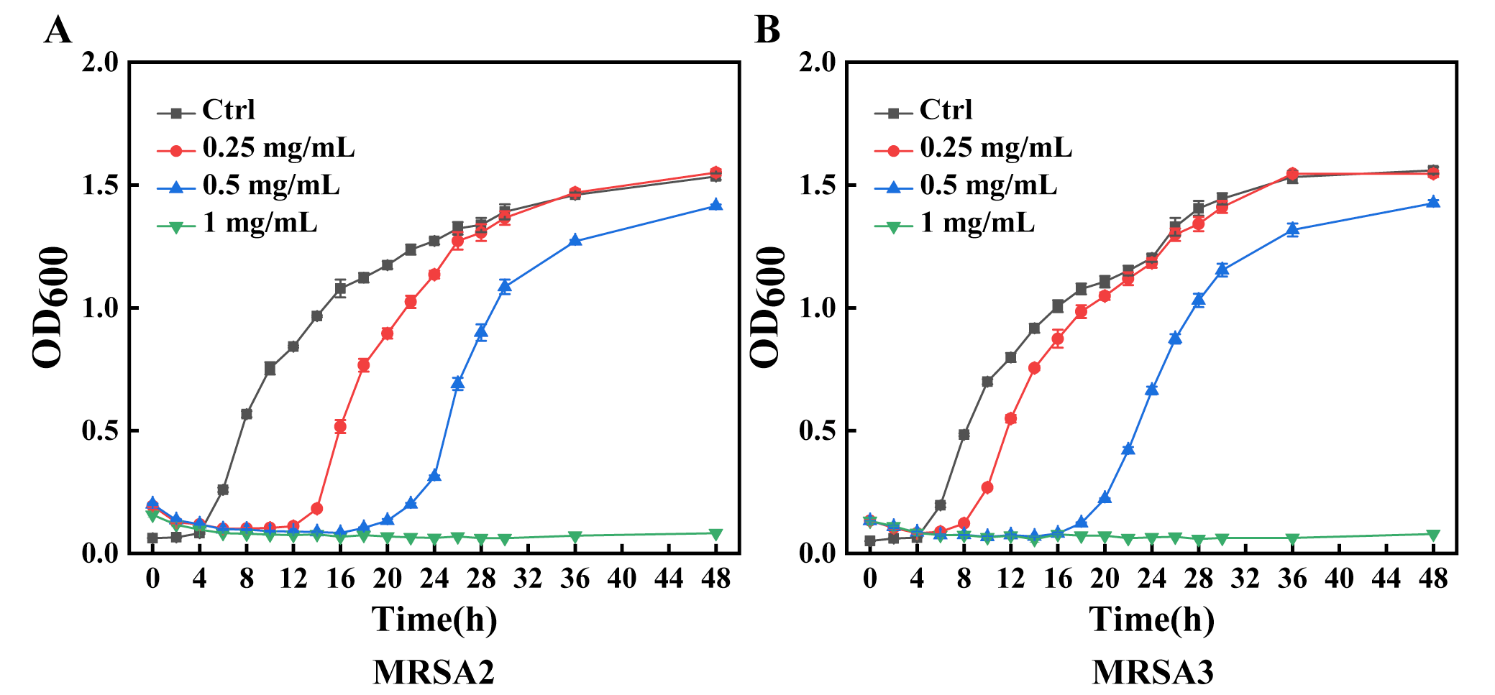


**Supplementary Figure 3.** Curve graph of the effect of HBPL on the growth of MRSA2 (A) and MRSA3 (B).


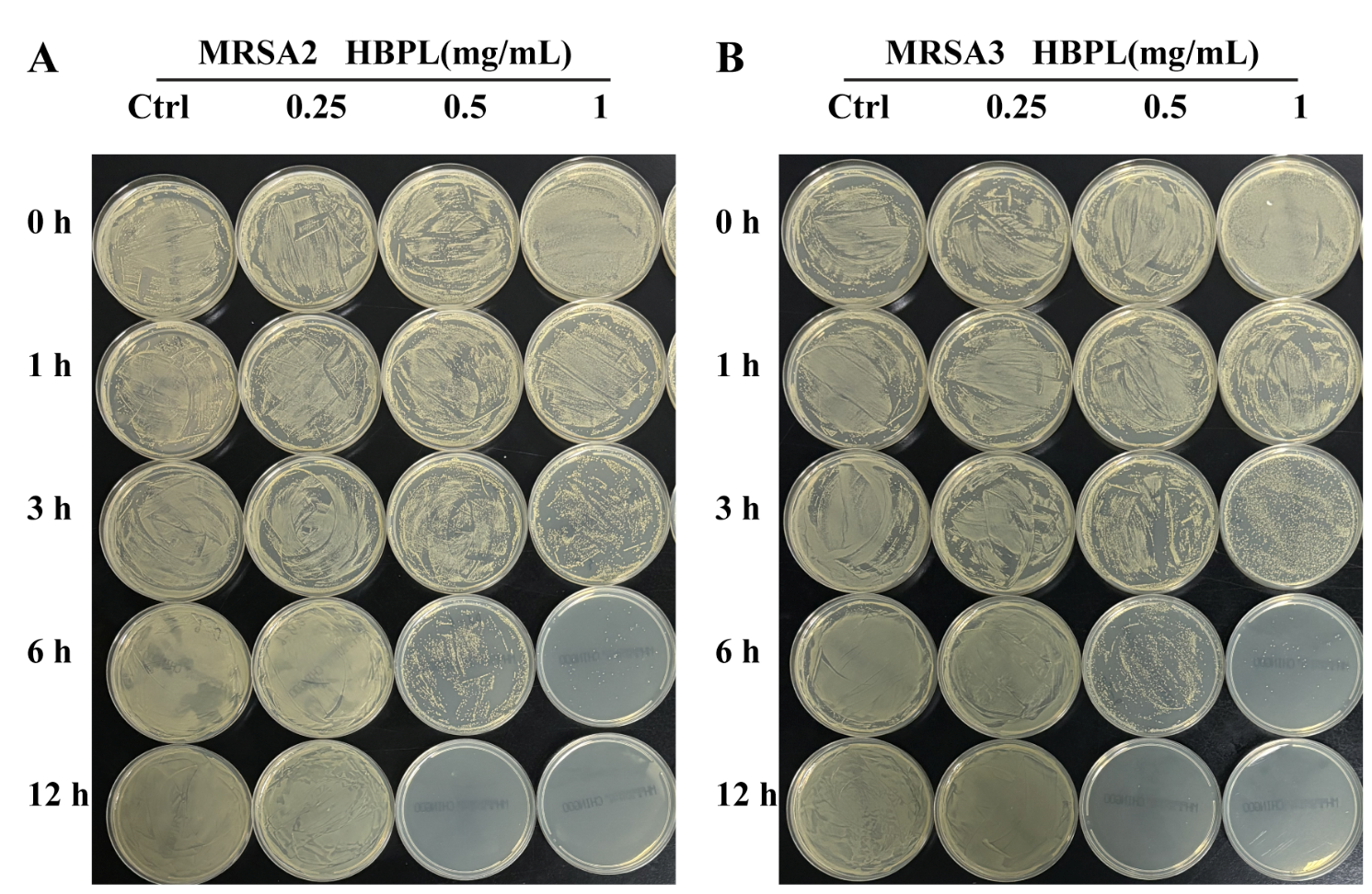


**Supplementary Figure 4.** The time and concentration dependence of HBPL on the killing of MRSA2 (A) and MRSA3 (B).


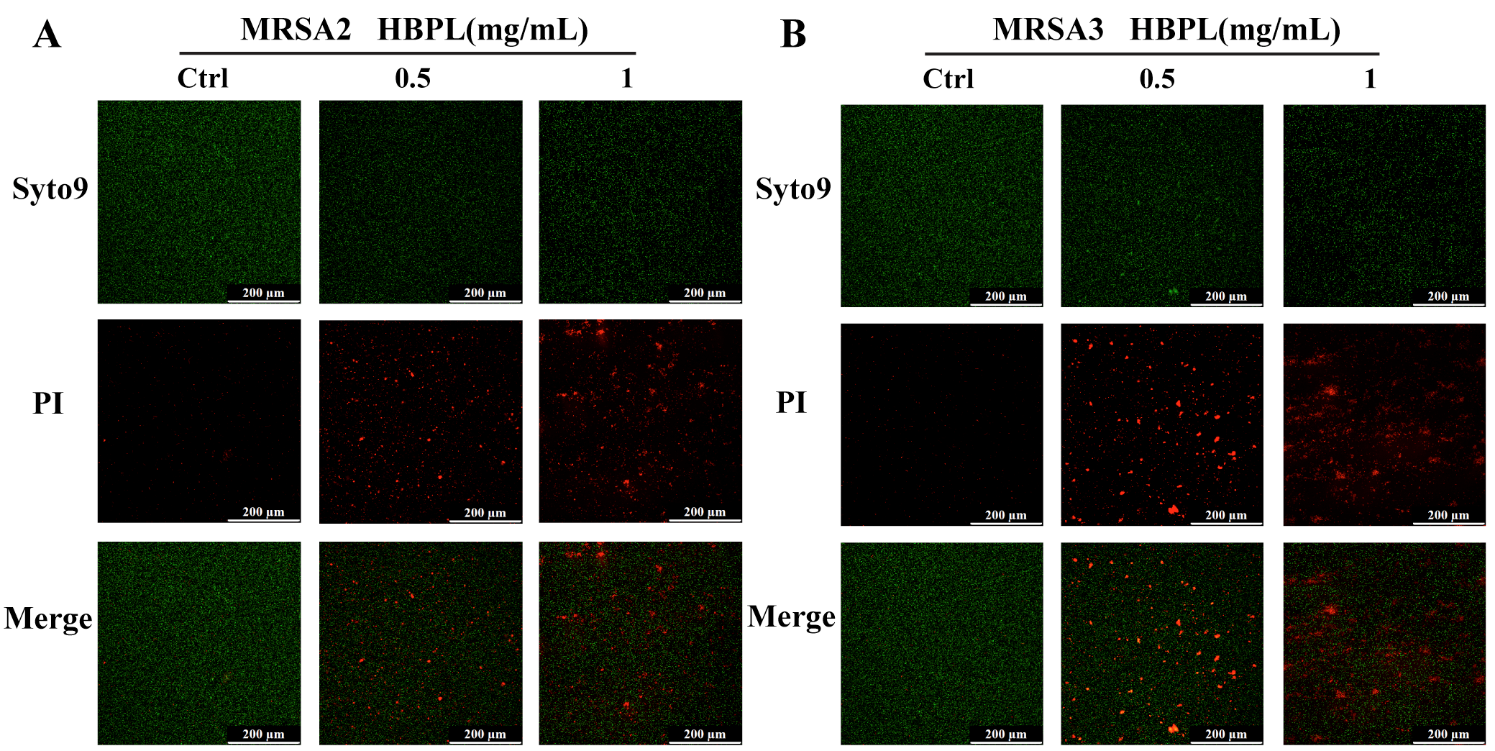


**Supplementary Figure 5.** Fluorescence microscopy images of MRSA2 (A) and MRSA3 (B) treated with different concentrations of HBPL.


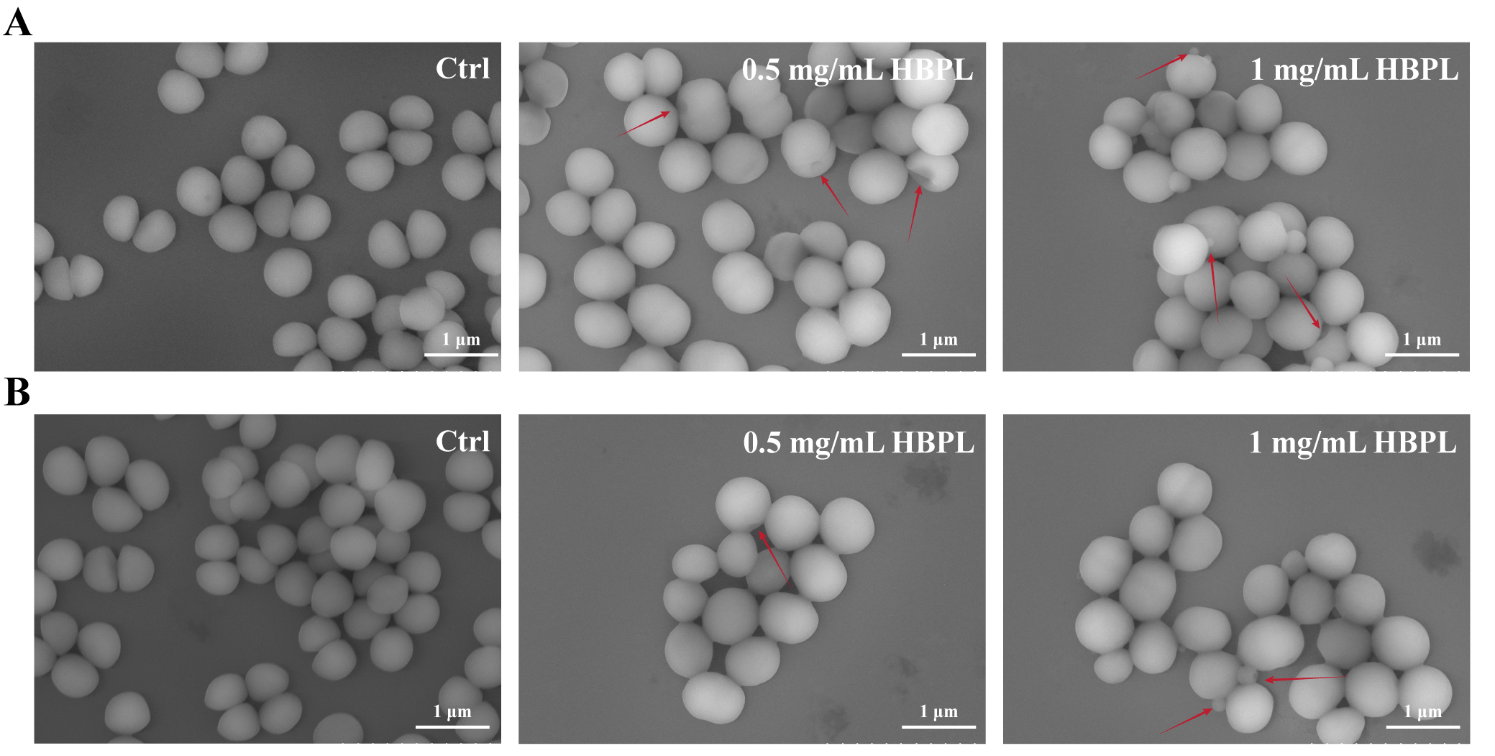


**Supplementary Figure 6.** Scanning electron microscopy images of MRSA2 (A) and MRSA3 (B) treated with different concentrations of HBPL.


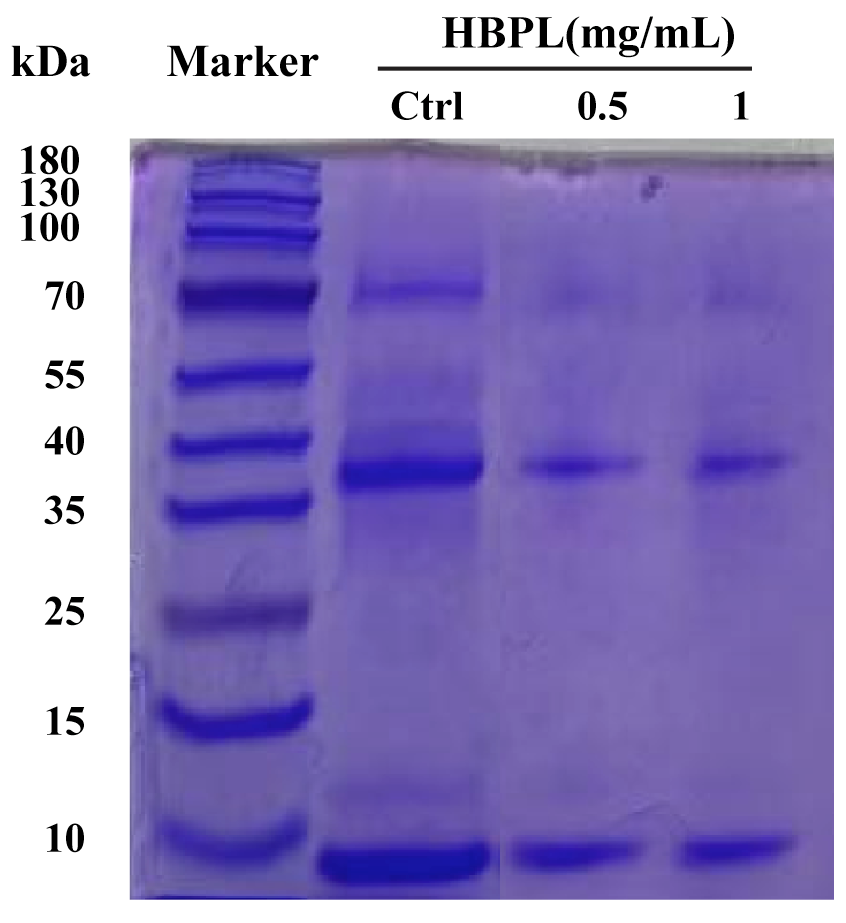


**Supplementary Figure 7.** The effect of HBPL on the protein spectrum of MRSA1.

**
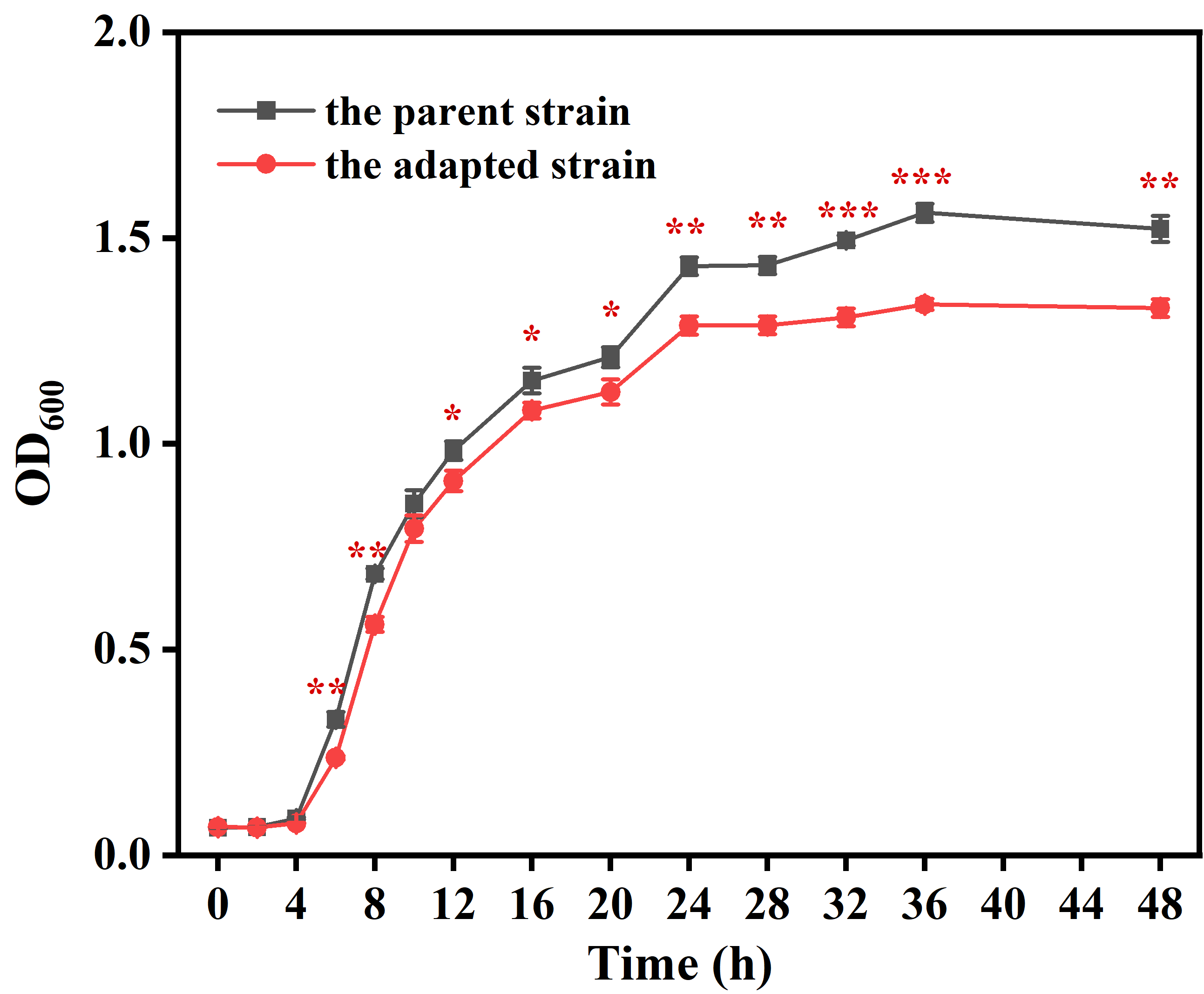
**

**Supplementary Figure 8.** Growth curve of the parent strain and the HBPL-adapted strain in HBPL-free medium. Each experiment was in three technical replicates. Error bars represent standard error of the mean. **p* < 0.05; ***p* < 0.01, ****p* < 0.001.

| Time(d) | 0 | 1-4 | 5-8 | 9-12 | 13-16 | 17-20 | 21-24 | 25-28 |
| --- | --- | --- | --- | --- | --- | --- | --- | --- |
| Experimental group | 0 | 0.25 | 0.5 | 1 | 2 | 4 | 8 | 0 |
| Ctrl | 0 | 0 | 0 | 0 | 0 | 0 | 0 | 0 |

**Supplementary Table 1.** Daily HBPL concentration (mg/mL) in each group.
